# Are social pressure, bullying and low social support associated with depressive symptoms, self-harm and self-directed violence among adolescents? A cross-sectional study using a structural equation modeling approach

**DOI:** 10.1186/s12888-024-05696-1

**Published:** 2024-03-29

**Authors:** Tonje Holte Stea, Tore Bonsaksen, Pierre Smith, Annette Løvheim Kleppang, Anne Mari Steigen, Marja Leonhardt, Lars Lien, Mario Vianna Vettore

**Affiliations:** 1 Department of Health and Nursing Science, University of Agder, Kristiansand, Norway; 2 Department of Health and Nursing Sciences, Inland Norway University of Applied Sciences, Elverum, Norway; 3 Department of Health, VID Specialized University, Stavanger, Norway; 4 Health information service. Epidemiology and public health. Sciensano, Brussels, Belgium; 5 Institute of Health and Society, Université catholique de Louvain, Brussels, Belgium; 6 Department of Public Health and Sport Sciences, Inland Norway University of Applied Sciences, Elverum, Norway; 7 Norwegian National Advisory Unit on Concurrent Substance Abuse and Mental Health Disorders, Innlandet Hospital Trust, Brumunddal, Norway; 8 Department of Dentistry and Oral Health, Aarhus University, Aarhus, Denmark

**Keywords:** Adolescent, Social pressure, Bullying, Social support, Depressive symptoms, self-harm, Suicide thoughts

## Abstract

**Background:**

More in-depth evidence about the complex relationships between different risk factors and mental health among adolescents has been warranted. Thus, the aim of the study was to examine the direct and indirect effects of experiencing social pressure, bullying, and low social support on mental health problems in adolescence.

**Methods:**

A school-based cross-sectional study was conducted in 2022 among 15 823 Norwegian adolescents, aged 13–19 years. Structural Equation Modelling was used to assess the relationships between socioeconomic status, social pressure, bullying, social support, depressive symptoms, self-harm and suicide thoughts.

**Results:**

Poor family economy and low parental education were associated with high pressure, low parental support and depressive symptoms in males and females. Moreover, poor family economy was associated with bullying perpetration and bullying victimization among males and females, and cyberbullying victimization among females, but not males. Low parental education was associated with bullying victimization among males, but not females. Further, high social pressure was associated with depressive symptoms among males and females, whereas high social pressure was linked to self-harm and suicide thoughts among females, but not males. Bullying victimization and cyberbullying victimization were associated with depressive symptoms, self-harm, and suicide thoughts among males and females. Bullying victimization was associated with depressive symptoms among males, but not females, whereas bullying perpetration was linked to self-harm and suicide thoughts among females, but not males. Low parental support was associated with bullying perpetration, bullying victimization, depressive symptoms, self-harm and suicide thoughts among males and females, whereas low parental support was associated with high social pressure among females, but not males. Low teacher support was associated with high social pressure and depressive symptoms. Low support from friends was associated with bullying victimization, depressive symptoms and suicide thoughts among males and females, whereas low support from friends was linked to self-harm among males, but not females. Finally, results showed that depressive symptoms were associated with self-harm and suicide thoughts among males and females.

**Conclusion:**

Low socioeconomic status, social pressure, bullying and low social support were directly and indirectly associated with depressive symptoms and self-directed violence among Norwegian adolescents.

**Supplementary Information:**

The online version contains supplementary material available at 10.1186/s12888-024-05696-1.

## Background

Depression and self-directed violence have been identified as major public health problems among youth in the general population. According to the World Health Organization (WHO), depression is the fourth leading cause of illness and disability, and self-harm causes 256,180 deaths globally per year among adolescents and young adults [1, 2]. Moreover, depressive symptoms continue to increase, especially among females [3–5]. In the UK, self-harm incidence rate among girls aged 13–16 years increased substantially during the COVID-19 pandemic in contrast to the rate seen in boys and participants in older age groups [6]. In Norway, a sharp increase in mental health problems and suicide risk was partly explained by less physical meetings on campus among both male and female students during the COVID-19 pandemic [7]. Moreover, a study among Norwegian adolescents reported that the level of depressive symptoms has increased markedly, and the prevalence of self-harm has increased 4-fold over a 15-year period, and that such increase in depressive symptoms could explain some of the increase in self-harm [8].

The identification of possible risk factors and protective factors are essential to prevent potentially long-lasting mental health problems among adolescents. Across most countries, more girls than boys are at risk of suicidal thoughts and related behaviors [9]. However, studies have identified “the gender paradox in suicide,” in which non-fatal suicidal behavior is more common among females, whereas males are overrepresented among those who died by suicide [10, 11]. A comprehensive Swedish population-based cohort study among adolescents has also confirmed the association between low parental socioeconomic status and self-harm, predominantly among females [12].

Adolescence is characterized by increased stress sensitivity [13], and adolescents who present higher levels of perceived stress have shown increased risk of suicidal behavior [14] Other studies have confirmed the association between social pressure to perform in school and experiencing academic stress and increased psychological symptoms [15, 16], and a negative but small impact of social media use on adolescent mental health [17, 18]. Moreover, body dissatisfaction and weight discrimination has been associated with increased risk of self-harm and suicide ideation [19, 20]. For most adolescents, participation in sports have demonstrated a beneficial influence on mental health, reducing the risk of depression and suicidal thoughts [21, 22]. However, greater social pressure to perform well and experiencing highly competitive environments may have negative psychological effects that may increase the total burden of stress [23, 24].

A recent cohort study among Norwegian adolescents showed that increased stress, including experience of stressful life events and bullying victimization at age 12, were associated with increased depression at age 14 more strongly among females than males [25]. A new review highlighted the key role of depression as a mediator of the relationship between exposure to traditional / cyber victimization and self-harmful thoughts and behavior in young people [26]. In addition, females involved in bullying were at higher risk for suicide [26]. A study among Welsh adolescents also supported the role of in-person bullying victimization on self-harm, as those who had experienced in-person bullying were twice as likely to self-harm than their non-victimized peers [27]. However, it has been suggested that the complexity and severity of the consequences of bullying are dependent on the intensity and duration of the exposure that interact with a range of risk and protective factors [28].

Adolescence is a period of life characterized by increased sensitivity to social stimuli and increased need for interaction with peers [29], and higher perceived social support has been identified as one of the most important protective factors of adolescents’ mental health problems, irrespective of gender [30]. Although support from friends in adolescence has been identified as an important promoter of positive mental health in early adulthood, parental support and perceived support from teachers have also been positively and prospectively associated with meaningfulness [31]. Other studies have shown that an increase in social support from parents and peers reduced symptoms of psychological distress due to academic problems [32], and that social support from friends seemed to counteract the link between continued bullying victimization from childhood to young adulthood and subsequent depressive symptoms [33]. A qualitative study also concluded that interpersonal relationships appear to be crucial for assisting adolescents in coping with stressors, acting as sources of social support that protect against psychological distress [34].

Social-ecological theory and research emphasize the multiple aspects of a child’s life that interacts with and affects the child, and that their behavior depends on the reciprocal interaction of personal, behavioral, and environmental factors [35]. The social causation hypothesis posits that socio-economic hardship, including financial stress and decreased social capital, increases the risk of mental illness [36]. Moreover, the strain theory of suicide emphasizes that strains, resulting from conflicting and competing pressures in an individual’s life, are hypothesized to precede suicide, whereas higher social support may mitigate strains and reduce their impact on suicidal behavior [37, 38]. Using these theories as a conceptual framework for the present study, we hypothesized that poor family economy, low parental education, and low social support from friends, teachers and parents would be directly associated with higher social pressure, more involvement in bullying (bullying victimization or bullying perpetration), depressive symptoms, self-harm, and suicide thoughts. In addition, self-harm and suicide thoughts would be directly associated with higher social pressure, more involvement in bullying and depressive symptoms.

Hence, we aimed to provide more in-depth evidence about the complex relationships between multiple risk factors and mental health among adolescents. We investigated the relationships between socioeconomic characteristics, different forms of social pressure, bullying, social support, and mental health outcomes including depressive symptoms, self-harm, and suicide thoughts among Norwegian adolescents.

## Methods

### Data source

Ungdata (Young data) is a national data collection scheme designed to conduct cross-sectional surveys of adolescents aged between 13 and 19 years. The survey is usually conducted every three years at the municipality level in Norway. More information on Ungdata is available elsewhere (www.ungdata.no). The survey collects data on various aspects of young people’s lives and is regarded as the most comprehensive source of information on adolescent health and well-being at the municipal and national levels in Norway. It is among other things used in municipal planning and developmental work related to public health and preventive measures aimed at young people.

### Study participants and data collection

The present study was conducted in 2022 among adolescents attending junior and senior high schools in southern Norway. In total, 22,452 (10,664 junior high school) students were invited to participate. Of these, 16,181 students agreed (junior high school students: *n* = 8,929, high-school students: *n* = 7,252), yielding a participation rate of 75% among the total sample (90% among junior high students and 68% among high school students, respectively). A total of 8,708 junior high school students and 7,115 high school students (total *n* = 15,823) completed the self-administered questionnaire and were included in the present study. Background characteristics of the participants indicated somewhat higher parental educational level compared to national registers [39].

The participants spent approximately 30 min to complete the online self-administered questionnaire during school hours. At least one member of the project group was present during the data-collection to clarify any doubts.

### Measures

The questionnaire included questions about sociodemographic factors, bullying, social pressure, different types of social support, depressive symptoms, self-harm, and suicide thoughts.

#### Self-directed violence

Information about exposure to self-directed violence was retrieved by asking the following questions: “Have you ever thought about taking your own life?” and “Have you ever tried to harm yourself, but without the intention to die?”. Response alternatives for both questions were (1) “No”, (2) “Yes, once”, and (3) “Yes, several times”. For descriptive analyses, self-directed violence was identified by combing the response options “Yes, once” and “Yes, several times” [1] and compared to those with no exposure to self-directed violence (0).

#### Depressive symptoms

Depressive symptoms were measured using a six items scale derived from the Hopkins Symptom Checklist [40, 41]. Adolescents were asked if during the past week they experienced any of the following: “Felt that everything is a struggle” (item 1), “Had sleep problems” (item 2), “Felt unhappy, sad or depressed” (item 3), “Felt hopelessness about the future” (item 4), “Felt stiff or tense” (item 5), “Worried too much about things” (item 6). Participants respond on a scale from 1 to 4 as follows: 1) ”Not been affected at all”, 2),“not been affected much”, 3) “been affected quite a lot”, and 4) “been affected a great deal”. The responses were summarized across all items which resulted in a sum score ranging from 6 to 24. Higher scores indicated higher levels of depressive symptoms. To capture depressive symptoms for presentation in descriptive analyses, we dichotomized the variables by using a cut-off at 3 to classify participants with average scores of quite distress or higher [1] and those with lower scores (0). The depressive-symptom scale has been evaluated psychometrically among Norwegian adolescents and has demonstrated good reliability (Person Separation Index: 0.802), and on a general level, the scale works reasonably well [42].

*Sociodemographic variables* Information about the participant’s sex was retrieved by asking participants whether they were male or female. Grade level was applied as a proxy for adolescent age.

Parent’s educational level was assessed by asking respondents whether their mother and/or father had a completed college/university education. The response categories were “no” or “yes” (high educational level as the reference category) for both the maternal and paternal educational levels. The responses to questions reflecting maternal and paternal education were collapsed and presented as low [1], medium [2] and high [3] parental education, in which high educational level for both parents was classified as high parental education. Perceived family economy was based on responses to the question: “Financially, has your family been well off or badly off, over the past two years”? The response options were: (1) “We have been well off the whole time”, (2) “We have generally been well off”, (3) “We have neither been well off or badly off” (4) “We have generally been badly off” and (5) “We have been badly off the whole time”.

#### Social pressure

Social pressure was a latent variable supported by four indicators: body/looks, school performance, sports performance, and followers/likes on social media. The scores of four items were used as indicators: “Do you feel pressure in your everyday life to look good or have a nice body”, “Do you feel pressure in your everyday life to perform well at school”, “Do you feel pressure in your everyday life to perform well in sports”, “Do you feel pressure in your everyday life to have many followers and likes on social media”. Response options were: (1) “No pressure”, (2) “A little pressure”, (3) “Some pressure”, (4) “A lot of pressure”, and (5) “Very much pressure”. The response alternatives “Very much pressure” and “A lot of pressure” were used to identify high [1] versus low (0) pressure for presentation in descriptive analyses. Cronbach’s alpha for social pressure scale was 0.72 for boys and 0.73 for girls.

#### Bullying

Bullying was measured by two questions reflecting bullying victimization and one question reflecting bullying perpetration. Bullying victimization was measured by the following questions: “Are you sometimes teased, threatened, or frozen out by other young people in school or in your free time?” and “Are you sometimes teased, or threatened, by other young people online or on your mobile phone?” Bullying perpetration was measured by the following question: “Do you sometimes take part in teasing, threatening or freezing out other young people at school or in your free time?”. The response options for the questions were: (1) “Yes, several times a week”, (2) “Yes, approximately once a week”, (3) “Yes, approximately once every fortnight”, (4) “Yes, approximately once a month”, (5) “Almost never”, and (6) “Never”. For descriptive analyses, bullying behavior was identified by dichotomizing the response options into once or several times/week [1] or less often/never (0).

#### Social support

Social support from friends was composed of three indicators: friends to trust, friends available after school, and friends at school. Information about teacher and parental support was based on responses to the questions: “Do the teachers care about me?” and “Are my parents very interested in my life?”. Response alternatives for both questions were: (1) “Totally agree”, (2) “Somewhat agree”, (3) “Somewhat disagree”, and (4) “Totally disagree”. The response alternative “Totally agree” was used to identify participants with stronger perceived social support from teacher and parents (high support) for presentation in descriptive analyses.

Social support from friends was a latent variable measured by three indicators: friends to trust, friends available after school, and friends at school. The scores of the following three items were used to assess the indicators: “Do you have at least one friend who you trust completely and can tell absolutely anything?” (Response options: 1) “Yes, I am sure”, 2) “Yes, I think so”, 3) “I do not think so”, and 4) “I have no one I could call a friend right now”), “Do you have someone to spend time with after school hours?” and, “Do you have someone to spend time with during breaks at school?”. Response options for questions 2 and 3 were: (1) “Yes, all the time”, (2) “Yes, most of the time”, (3) “No, mostly not”, and (4) “No, never”. For question 1, response options “Yes, I am sure” and “Yes, I think so” were used to identify participants with friends to trust [1] and those without friends to trust (0). For question 2 and 3, response options “Yes, all of the time” and “Yes, most of the time” were used to identify participants with friends available after school or at school [1] and those without friends available after school or at school (0). Cronbach’s alpha for social support from friend’s scale was 0.65 for boys and 0.66 for girls.

### Statistics

Descriptive analyses reported the distribution of the variables through proportions, and differences according to sex was examined using Pearson’s chi-square test. Confirmatory factorial analysis (CFA) and Structural equation modelling (SEM) were conducted separately for females and males when testing the associations between variables. The former was conducted to evaluate the measurement model involving the two latent variables (social pressure and social support from friends). SEM was used to examine the direct and indirect relationships between observed and latent variables according to the conceptual framework (Appendix 1). The standardized direct effects represent a direct path from one variable to another, and standardized indirect associations indicate a path between two or more variables mediated by another variable. Parental educational levels and perceived family economy were included in the SEM for adjustment. The Maximum likelihood estimation methods was used to estimate the standardized total, direct and indirect effects, and the standard errors and 95% confidence intervals (95% CIs) were used to assess mediation by analyzing the statistical significance of indirect associations [43]. After estimating the full model (see Appendix 1), non-significant direct paths were removed, and a statistically parsimonious model was re-estimated. The adequacy of the measurement and structural models was evaluated according to the following fit indices and threshold values: standardized root mean square residual (SRMR) ≤ 0.08, comparative fit index (CFI) ≥ 0.90, goodness of fit index (GFI) ≥ 0.90 and root-mean-square error of approximation (RMSEA) ≤ 0.06 [40]. The significance level established for all analyses was 5% (*p* < 0.05). All analyses were performed using statistical software STATA 17.0 (StataCorp, College Station, TX, USA).

## Results

Descriptive characteristics according to sex are presented in Table 1. The distribution of participating males and females varied according to school level: a higher number of the males attended junior high school than senior high school compared to females. Parental educational level did not vary according to sex, but a higher proportion of males reported good family economy compared to females. Moreover, a higher proportion of females than males reported to experience high social pressure related to body/looks, school- and sports performance and having followers/likes on social media. Further, a higher proportion of males than females reported bullying perpetration, whereas more females than males reported symptoms of depression, self-harm, suicide thoughts and suicide attempts. Finally, a higher proportion of males reported to receive high social support from teachers and from friends, both during and after school hours.

**Table 1:** Descriptive statistics, Norwegian females (*n* = 8380) and males (*n* = 8102), 2022

	Females n (%)	Males n (%)	*p*-value*
**School level**			
Junior high school	4282 (53.3)	4426 (56.8)	< 0.001
High school	3754 (46.7)	3361 (43.2)	
**Parental education**			
High, both parents	3947 (51.0)	3775 (51.7)	0.446
**Family economy**			
Good family economy, all the time	3447 (42.6)	3655 (47.6)	< 0.001
**High pressure**			
Body/looks	3009 (38.0)	671 (9.4)	< 0.001
School performance	3756 (47.3)	1555 (21.9)	< 0.001
Sports performance	1791 (22.8)	900 (12.7)	< 0.001
Followers/likes on social media	716 (9.0)	163 (2.3)	< 0.001
**Bullying**			
Bullied others, ≥ once/week	94 (1.2)	221 (2.9)	< 0.001
Bullied by others, ≥ once/week	343 (4.2)	332 (4.4)	0.656
Bullied by others online, ≥ once/week	159 (2.0)	179 (2.3)	0.090
**Support by friends**			
Friends to trust	5057 (61.8)	4714 (60.9)	0.267
Friends available after school	3874 (47.3)	4042 (52.2)	< 0.001
Friend at school	5617 (68.6)	5815 (75.1)	< 0.001
**Teacher support**			
High support by teacher	2900 (35.6)	3225 (41.9)	< 0.001
**Parental support**			
High parental support	4384 (54.0)	4101 (53.4)	0.483
**Depression**			
Symptoms of depression	678 (8.7)	212 (3.0)	< 0.001
**Self-harm**			
Yes, once or more	6565 (25.2)	740 (11.0)	< 0.001
**Suicide thoughts**			
Yes, once or more	2401 (31.6)	1303 (19.4)	< 0.001
**Suicide attempts**			
Yes, once or more	463 (6.1)	250 (3.7)	< 0.001

*Analyses were conducted using the Chi-square test

### Confirmatory factor analysis

Confirmatory factor analysis (CFA) supported the presence of individual latent factors ‘social pressure’ and ‘social support from friends’ for females and males (Appendix 2). The item loadings confirming the latent variables were 0.534 or higher for females and 0.470 or higher for males.

### Structural equation modelling

SEM supported the hypothesized (full), measurement and parsimonious models for females and males with the values: CFI ≥ 0.927, SRMR ≤ 0.055, RMSEA ≤ 0.057 (Appendix 3).

### Direct effects

**Fig. 1:**
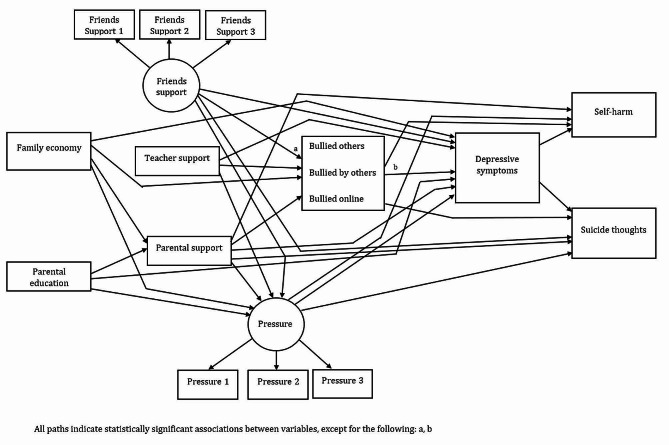
Parsimonious model of associations between the predictors of depressive symptoms, self-harm and suicide thoughts for females

**Fig. 2:**
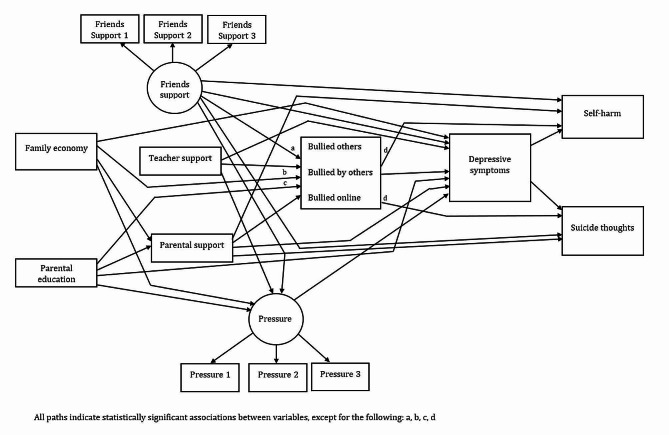
Parsimonious model of associations between the predictors of depressive symptoms, self-harm and suicide thoughts for males

All the direct effects reported in the parsimonious models for females (Fig. 1; Table 2) and males (Fig. 2; Table 3) were in the expected direction.

**Table 2:** Standardized direct effects of the parsimonious structural equation model on the relationships between the predictors of depressive symptoms, self-harm and suicide thoughts for females

Variables	Self-harm		
	β	95% CI	*p*-value
Pressure, high	0.07	0.04/0.10	< 0.001
Bullied others, low	-0.03	-0.05/-0.01	0.012
Bullied by others, low	-0.09	-0.11/-0.06	< 0.001
Bullied online, low	-0.07	-0.10/-0.05	< 0.001
Parental support, low	0.12	0.10/0.14	< 0.001
Depressive symptoms, high	0.32	0.29/0.34	< 0.001
	**Suicide thoughts**		
Pressure, high	0.05	0.02/0.08	0.001
Bullied others, low	-0.06	-0.08/-0.04	< 0.001
Bullied by others, low	-0.04	-0.07/-0.02	0.001
Bullied online, low	-0.05	-0.08/-0.03	< 0.001
Friends support, low	0.07	0.04/0.10	< 0.001
Parental support, low	0.13	0.11/0.15	< 0.001
Depressive symptoms, high	0.41	0.39/0.44	< 0.001
	**Depressive symptoms**		
Family economy, low	0.11	0.09/0.13	< 0.001
Parental education, high	-0.05	-0.07/-0.03	< 0.001
Pressure, high	0.44	0.42/0.47	< 0.001
Bullied by others, low	-0.04	-0.07/-0.02	0.001
Bullied online, low	-0.04	-0.07/-0.02	< 0.001
Friends support, low	0.15	0.12/0.18	< 0.001
Teacher support, low	0.14	0.12/0.16	< 0.001
Parental support, low	0.12	0.09/0.14	< 0.001
	**Bullied others**		
Family economy, low	-0.05	-0.07/-0.02	< 0.001
Teacher support, low	-0.12	-0.14/-0.10	< 0.001
Parental support, low	-0.07	-0.10/-0.05	< 0.001
	**Bullied by others**		
Family economy, low	-0.04	-0.06/-0.01	0.004
Friends support, low	-0.37	-0.39/-0.34	< 0.001
Teacher support, low	-0.09	-0.11/-0.06	< 0.001
Parental support, low	-0.03	-0.06/-0.01	0.013
	**Bullied online**		
Family economy, low	-0.05	-0.08/-0.03	< 0.001
Friends support, low	-0.33	-0.36/-0.30	< 0.001
Teacher support, low	-0.10	-0.13/-0.08	< 0.001
Parental support, low	-0.04	-0.06/-0.01	0.003
	**Pressure**		
Family economy, low	0.08	0.05/0.01	< 0.001
Parental education, high	0.03	0.01/0.06	0.030
Friends support, low	0.21	0.17/0.24	< 0.001
Teacher support, low	0.25	0.22/0.27	< 0.001
Parental support, low	0.07	0.04/0.10	< 0.001
	**Parental support**		
Family economy, low	0.26	0.24/0.29	< 0.001
Parental education, high	-0.08	-0.11/-0.06	< 0.001

**Table 3:** Standardized direct effects of the parsimonious structural equation model on the relationships between the predictors of depressive symptoms, self-harm and suicide thoughts for males

Variables	Self-harm		
	β	95% CI	*p*-value
Bullied by others, low	-0.05	-0.08/-0.03	< 0.001
Bullied online, low	-0.10	-0.13/-0.07	< 0.001
Friends support, low	0.06	0.02/0.09	0.001
Parental support, low	0.05	0.03/0.08	< 0.001
Depressive symptoms, high	0.26	0.23/0.28	< 0.001
	**Suicide thoughts**		
Bullied by others, low	-0.05	-0.08/-0.03	< 0.001
Bullied online, low	-0.07	-0.09/-0.04	< 0.001
Friends support, low	0.07	0.03/0.10	< 0.001
Parental support, low	0.08	0.06/0.10	< 0.001
Depressive symptoms, high	0.46	0.44/0.48	< 0.001
	**Depressive symptoms**		
Family economy, low	0.07	0.05/0.09	< 0.001
Parental education, high	-0.05	-0.07/-0.03	< 0.001
Pressure, high	0.44	0.42/0.47	< 0.001
Bullied others, low	-0.04	-0.06/-0.10	0.005
Bullied by others, low	-0.04	-0.07/-0.01	0.004
Bullied online, low	-0.07	-0.09/-0.04	< 0.001
Friends support, low	0.17	0.14/0.20	< 0.001
Teacher support, low	0.11	0.09/0.14	< 0.001
Parental support, low	0.11	0.09/0.13	< 0.001
	**Bullied others**		
Family economy, low	-0.07	-0.09/-0.04	< 0.001
Teacher support, low	-0.14	-0.17/-0.11	< 0.001
Parental support, low	-0.06	-0.08/-0.03	0.013
	**Bullied by others**		
Family economy, low	-0.04	-0.06/-0.01	0.004
Parental education, high	-0.03	-0.05/-0.01	0.024
Friends support, low	-0.25	-0.28/-0.22	< 0.001
Teacher support, low	-0.09	-0.12/-0.07	< 0.001
Parental support, low	-0.05	-0.08/-0.02	< 0.001
	**Bullied online**		
Friends support, low	-0.29	-0.32/-0.26	< 0.001
Teacher support, low	-0.10	-0.12/-0.07	< 0.001
Parental support, low	-0.07	-0.09/-0.04	0.003
	**Pressure**		
Family economy, low	0.05	0.02/0.08	0.002
Parental education, high	0.05	0.02/0.08	0.002
Friends support, low	0.23	0.19/0.26	< 0.001
Teacher support, low	0.25	0.22/0.27	< 0.001
	**Parental support**		
Family economy, low	0.23	0.20/0.25	< 0.001
Parental education, high	-0.05	-0.07/-0.02	< 0.001

*Self-harm*: Bullying victimization (females: β = -0.09, males: β = -0.05), cyberbullying victimization (females: β = -0.07, males: β = -0.10), lower parental support (females: β = 0.12, males: β = 0.05), and depressive symptoms (females: β = 0.32, males: β = 0.26) were associated with self-harm in males and females. Higher social pressure (β = 0.07) and bullying perpetration (β = -0.03) were associated with self-harm among females, whereas lower support from friends (β = 0.06) was associated with self-harm in males.

*Suicide thoughts*: Bullying victimization (females: β = -0.04, males: β = -0.05), cyberbullying victimization (females: β = -0.05, males: β = -0.07), lower support from friends (females: β = 0.07, males: β = 0.07), lower parental support (females: β = 0.13, males: β = 0.08), and depressive symptoms (females: β = 0.41, males: β = 0.46) were associated with suicide thoughts in males and females. Higher social pressure (β = 0.05) and bullying perpetration (β = -0.06) were also linked to suicide thoughts among females.

*Depressive symptoms*: Poor family economy (females: β = 0.11, males: β = 0.07), lower parental education (females: β = -0.05, males: β = -0.05), higher social pressure (females: β = 0.44, males: β = 0.44), bullying victimization (females: β = -0.04, males: β = -0.04) and cyberbullying victimization (females: β = -0.04, males: β = -0.07), lower support from friends (females: β = 0.15, males: β = 0.17), lower teacher support (females: β = 0.14, males: β = 0.11), and lower parental support (females: β = 0.12, males: β = 0.11) were associated with depressive symptoms in males and females. Bullying perpetration was also linked to depressive symptoms among males (β = -0.04).

*Bullying perpetration*: Poor family economy (females: β = -0.05, males: β = -0.07), lower teacher support (females: β = -0.12, males: β = -0.14) and lower parental support (females: β = -0.07, males: β = -0.06) were directly linked to bullying perpetration among males and females.

*Bullying victimization*: Bullying victimization was related to poor family economy (females: β = -0.04, males: β = -0.04), lower support from friends (females: β = -0.37, males: β = -0.25), lower teacher support (females: β = -0.09, males: β = -0.09) and lower parental support (females: β = -0.03, males: β = -0.05). Lower parental education (β = -0.03) was also associated with bullying victimization among males.

*Cyberbullying victimization*: Lower support from friends (females: β = -0.33, males: β = -0.29), lower teacher support (females: β = -0.10, males: β = -0.10), lower parental support (females: β = -0.04, males: β = -0.07) were associated with cyberbullying victimization among males and females. Poor family economy was also linked to cyberbullying victimization among females (β = -0.05).

*Social pressure*: Poor family economy (females: β = 0.08, males: β = 0.05), lower parental education (females: β = 0.03, males: β = 0.05), lower support from friends (females: β = 0.21, males: β = 0.23) and lower teacher support (females: β = 0.25, males: β = 0.25) were associated with experiencing higher social pressure in males and females. Lower parental support was also linked to higher social pressure among females (β = 0.07).

*Parental support*: Poor family economy (females: β = 0.26, males: β = 0.23) and lower parental education (females: β = -0.08, males: β = -0.05) was associated with lower parental support in males and females.

### Indirect effects

There were a number of significant total indirect effects within the parsimonious models for females (Appendix 4) and males (Appendix 5). Self-harm and suicide thoughts were indirectly associated with family economy, parental education, social pressure, bullying victimization, cyberbullying victimization, support from friends, teacher support and parental support in females and males. Bullying perpetration was also indirectly associated with self-harm and suicide thoughts among males. Family economy and parental education were indirectly associated with depressive symptoms, bullying perpetration, bullying victimization and cyberbullying victimization in females and males. Family economy and parental education were indirectly associated with social pressure among females. Depressive symptoms were indirectly associated with support from friends, teacher support and parental support in females and males. These are the total indirect effects and are made up of a number of specific indirect paths.

Specific indirect paths between non-adjacent variables were calculated through the multiplication of standardized beta coefficients estimated in the direct paths in the parsimonious model among females (Appendix 6) and males (Appendix 7). Social pressure, bullying and depressive symptoms were the main factors affecting the associations between family economy, parental education, support from friends, teacher support and parental support, and self-harm and suicide thoughts.

## Discussion

### Socio-economic status

Results from the present study indicate that perceived poor family economy was directly associated with lower parental support, higher social pressure, bullying perpetration, bullying victimization, and depressive symptoms. Further, perceived poor family economy was associated with cyberbullying victimization among females, but not among males. Moreover, lower parental education directly indicated lower parental support, higher social pressure and depressive symptoms. Moreover, lower parental education indicated also bullying victimization among males, but not among females.

The impact of socio-economic status (SES) on mental health outcomes such as depressive symptoms or self-harm in adolescence is in line with the social causation theory which states that socio-economic hardship increases the risk of subsequent mental illness [36]. Previously published literature has documented that lower SES was related to reduced parental capacity to invest in developmental inputs of their children [44], antisocial behavior [45] and poor psychological functioning of adolescents and their families [46]. A study among German children and adolescents also confirmed that those with low SES, were more likely exposed to multiple stressful events and were exposed to a higher risk of developing mental health problems [47]. Although our study showed indirect paths between both indicators of low SES and self-harm and suicide thoughts, other studies have demonstrated inverse associations between different indicators of SES and self-harm, especially among females [10, 48, 49]. A linear association between decreasing household income and self-harm has also been identified [50], and that lower SES during childhood is further associated with a higher risk of self-harm with suicidal intent [51].

### Social pressure

Our study revealed a direct association between being exposed to high social pressure and experiencing depressive symptoms in both sexes, and self-harm and suicide thoughts in females, but not males. Different forms of social pressure were revealed, ranging from social pressure on body image, social pressure to maintain high levels of academic and athletic performance, and social pressure to have positive reviews on social media.

Previous research found a relationship between body image and self-esteem among adolescents, and that body image and self-esteem were associated with self-harm behavior [52]. Moreover, an increase in perceived schoolwork pressure has been reported over time, especially among females in the highest grades [53], and high levels of academic stress has been related to suicidal ideation, with resilient students able to bounce back from academic challenges [54]. Adolescents also experience social pressure outside of school, i.e., related to participation in sport activities and use of social network sites. A review of systematic reviews reported that although organized sports participation has mainly reported positive associations with psychological and social variables, negative associations were also highlighted, characterized by social maladjustment and depression, and linked to very high levels of involvement [55]. In general, the tendency to internalize emotions, especially among females, may partly explain the increased risk for negative health-related outcomes as a result of high social pressure and stress [56].

### Bullying

Our results also indicated that bullying victimization and cyberbullying victimization was associated with depressive symptoms, self-harm, and suicide thoughts among males and females. Bullying perpetration, however, was associated with depressive symptoms among males, but not females and with self-harm and suicide thoughts among females, but not males.

Results from other studies have indicated that being exposed to stressful life events and bullying victimization increased the risk of engaging in mental health problems [23], non-suicidal self-injury [57], and suicide thoughts and attempts [58]. A study investigating trends and instigators among adolescent suicide in the US showed that bullying (either traditional or cyber) was the main factor associated with suicidal ideation, whereas the main factor associated with suicide attempt was sexual violence followed by physical bullying [59]. Our results in line with results presented in a systematic review indicating that depression mediates the relationship between traditional and cyber victimization and self-harmful thoughts and behavior in young people, and that females involved in bullying may potentially be at greater risk of suicide if depression is also present [24]. Thus, findings from the present study are in accordance with results from a national study among U.S. adolescents [60]. It is also in line with the model suggested by the general strain theory, with associations between bullying and suicidality being weakened when accounting for depressive symptoms [38].

### Social support

A systematic review on factors that influence the impact of cyberbullying on suicidal and self-harm behaviors reported that parental support was associated with less risk of suicidal and self-harm behaviors following cyberbullying [61]. In the present study, low parental support was linked to bullying perpetration, bullying victimization, cyberbullying victimization, depressive symptoms, self-harm and suicide thoughts among males and females, and high social pressure among females, but not males. We also investigated the possible role of teacher and peer support. Low teacher support was associated with high social pressure, depressive symptoms, whereas low friend’s support was linked with being bullied, depressive symptoms and suicide thoughts among males and females, and self-harm among males, but not females.

Results from another study among Norwegian adolescents, however, did not provide solid support for the buffering effects of social support related to stress of school performance on wellbeing, or anxiety and depression symptoms [62]. Findings from other studies also suggest that leveraging social support may play an important role in the prevention and treatment of mental health problems [63], and that parent and school support are relatively more important than peer support in understanding suicidal thoughts and history of suicidal behavior [64]. The longitudinal relationship between higher levels of perceived social support and fewer mental health problems suggest that perceived social support may protect against mental health problems during the transition into adulthood [63]. Thus, results from the present study and previously published results suggest that improving social support across these domains may be important in prevention and treatment of mental health problems and suicide attempts during this vulnerable transition period.

### Strength and weaknesses

Important strengths of the present study were that we analyzed different potential risk factors for depressive symptoms and self-directed violence according to gender in a large, nationally representative sample of Norwegian adolescents. To our knowledge, no previously published study has examined a wide range of risk factors of depressive symptoms, self-harm and suicide thoughts for this vulnerable group. In addition, we assessed the direct and indirect relationships between variable, which means the analyses of mediators. SEM was chosen as the statistical method since SEM simultaneously estimates complex relationships between multiple exposures (independent variables) and numerous outcomes (dependent variables). In addition, observable variables and unobservable variables (measured indirectly by multiple indicators through latent variables) are considered in the SEM. Finally, SEM accounts for measurement error in observed variables when estimating the relationships.

The following limitations of this study should be acknowledged. First, although SEM has increasingly been applied to cross-sectional studies, interpretations regarding directionality are limited and longitudinal studies are needed to understand the temporal unfolding of effects. Second, due to the cross-sectional design, we could not draw causal conclusions about the relationships between risk factors and adverse mental health outcomes as well as their mediating factors. Third, the present study relied on self-reported measures, and is therefore prone to suffer from memory and recall bias, as well as social-desirability bias. Finally, adolescents who were absent from school on the day of the data collection did not participate, and we could not assess whether the participants and nonparticipants differed in terms of the variables considered. However, we observed a somewhat higher parental educational level among the participants compared to national registers [39]. This finding may partly be explained by the lack of participants who have dropped out of school and recruitment of participants with higher social status.

## Conclusion

As proposed in our hypothesis, results from the present study showed several direct and indirect associations between different measures of socioeconomic status, social pressure, bullying, social support and adverse outcomes measured as depressive symptoms, self-harm and suicide thoughts in adolescents. These results are in line with the social causation theory, stating that socioeconomic hardship increases the risk of subsequent poor mental health. Therefore, this study highlights the importance of policies aiming at reducing economic and social inequalities, as they may also improve youth mental health. These results also provide increased knowledge about how multiple risk factors across domains impact both depressive symptoms, self-harm, and suicide thoughts among adolescents. More specifically, and in line with the strain theory of suicide, working on social determinants, such as social support, can be essential in preventing and treating these mental health problems. Thus, findings from the present study provide support for development of effective and targeted health promotion programs that specifically focus on improving mental health among male and female adolescents, respectively.

### Electronic supplementary material

Below is the link to the electronic supplementary material.

Supplementary Material 1

## Data Availability

Legal responsibility for the Young Data survey is held by the NOVA research center of Norwegian Social Research, Oslo and Akershus University College of Applied Sciences, which will provide information about data availability on reasonable request (ungdata@oslomet.no).
