# Supplementary material for: Are social pressure, bullying and low social support associated with depressive symptoms, self-harm and self-directed violence among adolescents? A cross-sectional study using a structural equation modeling approach
Source: BMC Psychiatry. 2024 Mar 29;24:239. doi: 10.1186/s12888-024-05696-1 (PMC10981317; doi:10.1186/s12888-024-05696-1)
Supplement: Supplementary file 1 — Supplementary Material 1 [file 12888_2024_5696_MOESM1_ESM.docx]

**Appendix 1.** Full theoretical model of the relationships between predictors and depressive symptoms, self-harm and suicide thoughts.


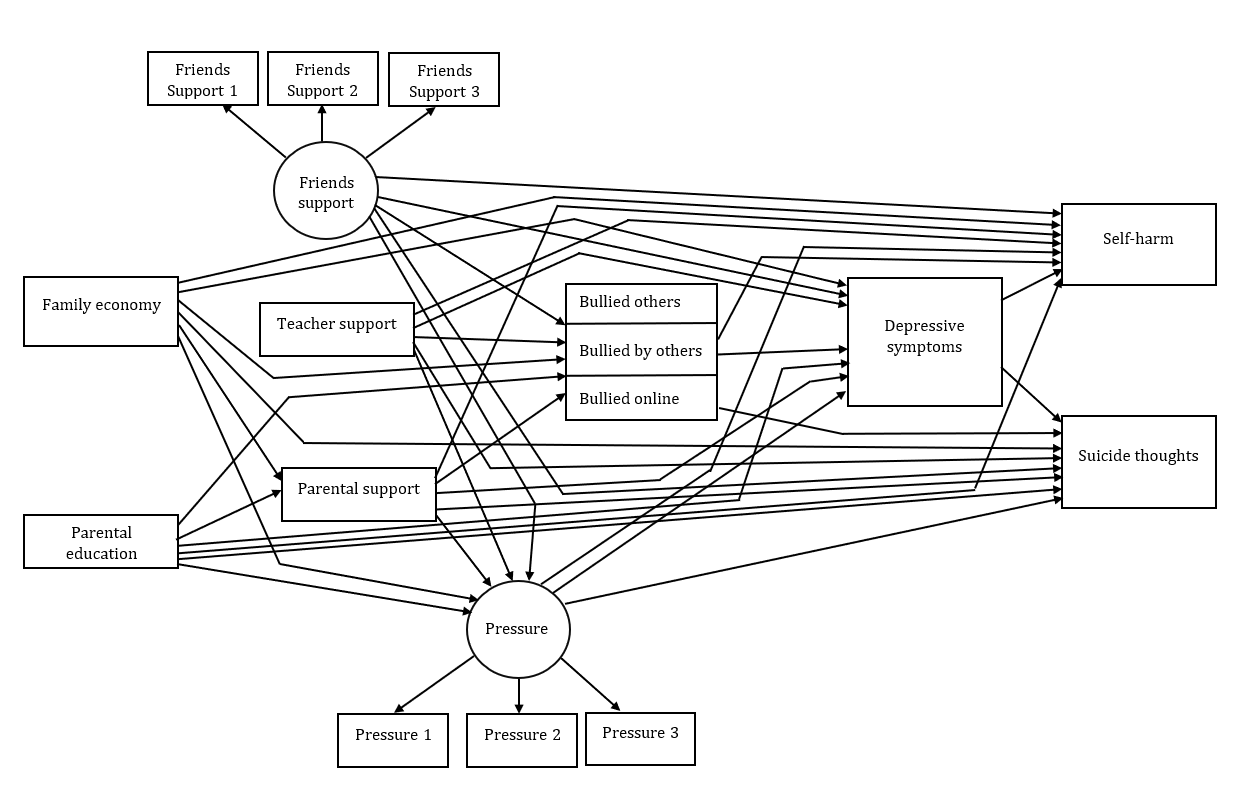


**Appendix 2.** Confirmatory factor analysis of the 2-factor 7 items (measurement model) for females and males.


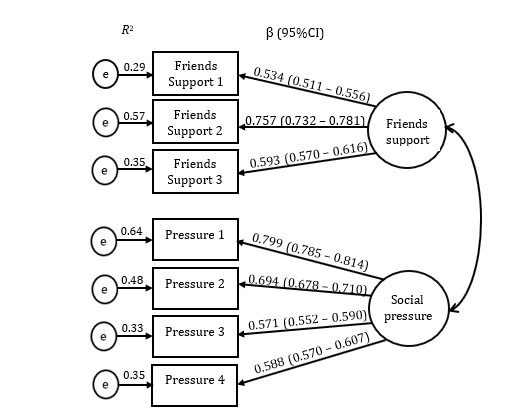


Females


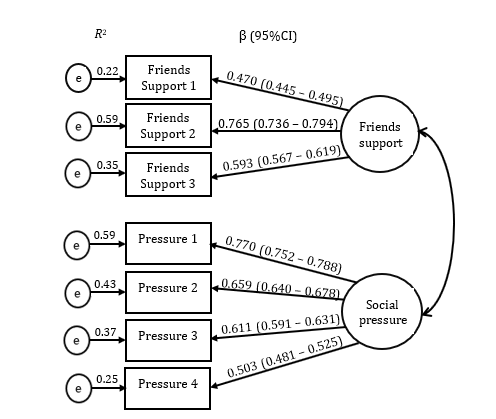


Males

**Appendix 3.** Fit indices for the confirmatory factor analysis of full, measurement and parsimonious models according to analysis by gender.

| **Model** | **CFI** | **SRMR** | **RMSEA** |
| --- | --- | --- | --- |
| ***Females*** |  |  |  |
| Full | 0.941 | 0.052 | 0.057 |
| Measurement | 0.975 | 0.024 | 0.053 |
| Parsimonious | 0.937 | 0.055 | 0.054 |
| ***Males*** |  |  |  |
| Full | 0.931 | 0.051 | 0.057 |
| Measurement | 0.973 | 0.027 | 0.050 |
| Parsimonious | 0.927 | 0.054 | 0.053 |

**Appendix 4.** Standardized indirect effects of the parsimonious structural equation model for females.

| Variables | Self-harm | |
| --- | --- | --- |
|  | β | p-value |
| Family economy, low | -0.11 | < 0.001 |
| Parental education, high | -0.02 | < 0.001 |
| Pressure, high | 0.14 | < 0.001 |
| Bullied by others, low | -0.01 | 0.001 |
| Bullied online, low | -0.01 | < 0.001 |
| Friends support, low | 0.16 | < 0.001 |
| Teacher support, low | 0.12 | < 0.001 |
| Parental support, low | 0.06 | < 0.001 |
|  | Suicide thoughts | |
|  | β | p-value |
| Family economy, low | 0.12 | < 0.001 |
| Parental education, high | -0.03 | < 0.001 |
| Pressure, high | 0.18 | 0.001 |
| Bullied by others, low | -0.02 | 0.001 |
| Bullied online, low | -0.02 | < 0.001 |
| Friends support, low | 0.16 | < 0.001 |
| Teacher support, low | 0.14 | < 0.001 |
| Parental support, low | 0.07 | < 0.001 |
|  | Depression | |
|  | β | p-value |
| Family economy, low | 0.08 | < 0.001 |
| Parental education, high | 0.00 | 0.876 |
| Friends support, low | 0.12 | < 0.001 |
| Teacher support, low | 0.12 | < 0.001 |
| Parental support, low | 0.03 | < 0.001 |
|  | Bullied others | |
|  | β | p-value |
| Family economy, low | -0.02 | < 0.001 |
| Parental education, high | 0.01 | < 0.001 |
|  | Bullied by others | |
|  | β | p-value |
| Family economy, low | -0.01 | 0.013 |
| Parental education, high | 0.00 | 0.019 |
|  | Bullied online | |
|  | β | p-value |
| Family economy, low | -0.01 | 0.003 |
| Parental education, high | 0.00 | 0.006 |
|  | Pressure | |
|  | β | p-value |
| Family economy, low | 0.02 | < 0.001 |
| Parental education, high | -0.01 | < 0.001 |

**Appendix 5.** Standardized indirect effects of the parsimonious model for males.

| Variables | Self-harm | |
| --- | --- | --- |
|  | β | p-value |
| Family economy, low | 0.05 | < 0.001 |
| Parental education, high | -0.01 | 0.007 |
| Pressure, high | 0.11 | < 0.001 |
| Bullied others, low | -0.01 | 0.006 |
| Bullied by others, low | -0.01 | 0.004 |
| Bullied online, low | -0.02 | < 0.001 |
| Friends support, low | 0.12 | < 0.001 |
| Teacher support, low | 0.07 | < 0.001 |
| Parental support, low | 0.04 | < 0.001 |
|  | Suicide thoughts | |
|  | β | p-value |
| Family economy, low | 0.08 | < 0.001 |
| Parental education, high | -0.02 | 0.005 |
| Pressure, high | 0.20 | 0.001 |
| Bullied others, low | -0.02 | 0.005 |
| Bullied by others, low | -0.02 | 0.004 |
| Bullied online, low | -0.03 | < 0.001 |
| Friends support, low | 0.17 | < 0.001 |
| Teacher support, low | 0.11 | < 0.001 |
| Parental support, low | 0.06 | < 0.001 |
|  | Depression | |
|  | β | p-value |
| Family economy, low | 0.05 | < 0.001 |
| Parental education, high | 0.02 | 0.020 |
| Friends support, low | 0.13 | < 0.001 |
| Teacher support, low | 0.11 | < 0.001 |
| Parental support, low | 0.01 | < 0.001 |
|  | Bullied others | |
|  | β | p-value |
| Family economy, low | -0.01 | < 0.001 |
| Parental education, high | 0.00 | 0.007 |
|  | Bullied by others | |
|  | β | p-value |
| Family economy, low | -0.01 | < 0.001 |
| Parental education, high | 0.00 | 0.010 |
|  | Bullied online | |
|  | β | p-value |
| Family economy, low | -0.02 | < 0.001 |
| Parental education, high | 0.00 | 0.003 |

**Appendix 6.** Calculation of specific indirect paths between variables for females.

***Self-harm***

***Family economy to self-harm***

Family economy → pressure → self-harm = 0.08 × 0.07 = 0.0056

Family economy → pressure → depression → self-harm = 0.08 × 0.44 × 0.32 = 0.011264

Family economy → bullied others → self-harm = -0.05 × -0.03 = 0.0015

Family economy → bullied by others → self-harm = -0.04 × -0.09 = 0.036

Family economy → bullied by others → depression → self-harm = -0.04 × -0.04 × 0.32 = 0.000512

Family economy → bullied online → self-harm = -0.05 × -0.07 = 0.0035

Family economy → bullied online → depression → self-harm = -0.05 × -0.07 × 0.32 = 0.00112

Family economy → parental support → self-harm = 0.26 × 0.12 = 0.0312

Family economy → parental support → bullied others → self-harm = 0.26 × -0.07 × -0.03 = 0.000546

Family economy → parental support → bullied by others → self-harm = 0.26 × -0.03 × -0.09 = 0.000702

Family economy → parental support → bullied by others → depression → self-harm = 0.26 × -0.03 × -0.04 × 0.32 = 0.00009984

Family economy → parental support → bullied online → self-harm = 0.26 × -0.04 × -0.07 = 0.000728

Family economy → parental support → bullied online → depression → self-harm = 0.26 × -0.04 × -0.04 × 0.32 = -0.000133

Family economy → parental support → pressure → self-harm = 0.26 × 0.07 × 0.07 = 0.001274

Family economy → parental support → pressure → depression → self-harm = 0.26 × 0.07 × 0.44 × 0.32 = 0.0025626

Family economy → depression → self-harm = 0.11 × 0.32 = 0.0352

***Parental education to self-harm***

Parental education → pressure → self-harm = -0.08 × 0.07 = -0.0056

Parental education → pressure → depression → self-harm = -0.08 × 0.44 × 0.32 = -0.011264

Parental education → parental support → self-harm = -0.08 × 0.12 = -0.0096

Parental education → parental support → bullied others → self-harm = -0.08 × -0.07 × -0.03 = -0.000168

Parental education → parental support → bullied by others → self-harm = -0.08 × -0.03 × -0.09 = -0.000216

Parental education → parental support → bullied by others → depression → self-harm = -0.08 × -0.03 × -0.04 × 0.32 = 0.000031

Parental education → parental support → bullied online → self-harm = -0.08 × -0.04 × -0.07 = -0.000224

Parental education → parental support → bullied online → depression → self-harm = -0.08 × -0.04 × -0.04 × 0.32 = -0.000041

Parental education → parental support → pressure → self-harm = -0.08 × 0.07 × 0.07 = -0.000392

Parental education → parental support → pressure → depression → self-harm = -0.08 × 0.07 × 0.44 × 0.32 = -0.000788

***Friends support to self-harm***

Friends support → pressure → self-harm = 0.21 × 0.07 = 0.0147

Friends support → pressure → depression → self-harm = 0.21 × 0.44 × 0.32 = 0.029568

Friends support → bullied by others → self-harm = -0.37 × -0.09 = 0.0333

Friends support → bullied by others → depression → self-harm = -0.37 × -0.04 × 0.32 = 0.004736

Friends support → bullied online → self-harm = -0.33 × -0.07 = 0.0231

Friends support → bullied online → depression → self-harm = -0.33 × -0.04 × 0.32 = 0.004224

Friends support → depression → self-harm = 0.15 × 0.32 = 0.048

***Teacher support to self-harm***

Teacher support → pressure → self-harm = 0.25 × 0.07 = 0.00175

Teacher support → pressure → depression → self-harm = 0.25 × 0.44 ×0.32 = 0.0352

Teacher support → bullied others → self-harm = -0.12 × -0.03 = 0.0036

Teacher support → bullied by others → self-harm = -0.09 × -0.09 = -0.0081

Teacher support → bullied by others → depression → self-harm = -0.09 × -0.04 × 0.32 = 0.001152

Teacher support → bullied online → self-harm = -0.10× -0.07 = 0.007

Teacher support → bullied online → depression → self-harm = -0.10 × -0.04 × 0.32 = 0.00128

***Parental support to self-harm***

Parental support → pressure → self-harm = 0.07 × 0.07 = 0.0049

Parental support → pressure → depression → self-harm = 0.07 × 0.44 × 0.32 = 0.009856

Parental support → bullied others → self-harm = -0.07 × -0.03 = 0.0021

Parental support → bullied by others → self-harm = -0.03 × -0.09 = 0.0027

Parental support → bullied by others → depression → self-harm = -0.03 × -0.04 × 0.32 = 0.000384

Parental support → bullied online → self-harm = -0.04 × -0.07 = 0.0028

Parental support → bullied online → depression → self-harm = -0.04 × -0.04 × 0.32 = 0.000512

Parental support → depression→ self-harm = 0.12 × 0.32 = 0.0384

***Pressure to self-harm***

Pressure → depression → self-harm = 0.44 × 0.32 = 0.1408

***Bullied by others to self-harm***

Bullied by others → depression → self-harm = -0.04 × 0.32= -0.0128

***Bullied online to self-harm***

Bullied online → depression → self-harm = -0.04 × 0.32 =-0.0128

***Suicide thoughts***

***Family economy to suicide thoughts***

Family economy → pressure → suicide thoughts = 0.08 × 0.05 =0.004

Family economy → pressure → depression → suicide thoughts = 0.08 × 0.44 × 0.41 = 0.014432

Family economy → bullied others → suicide thoughts = -0.05 × -0.06 = 0.003

Family economy → bullied by others → suicide thoughts = -0.04 × -0.04 = 0.0016

Family economy → bullied by others → depression → suicide thoughts = -0.04 × -0.04 × 0.41 = 0.000656

Family economy → bullied online → suicide thoughts = -0.05 × -0.05 = 0.0025

Family economy → bullied online → depression → suicide thoughts = -0.05 × -0.04 × 0.41 = 0.00082

Family economy → parental support → suicide thoughts = 0.26 × 0.13 = 0.0338

Family economy → parental support → bullied others → suicide thoughts = 0.26 × -0.07 × -0.06 = 0.001092

Family economy → parental support → bullied by others → suicide thoughts = 0.26 × -0.03 × -0.04 = 0.000312

Family economy → parental support → bullied by others → depression → suicide thoughts = 0.26 × -0.03 × -0.04 × 0.41 = 0.00012792

Family economy → parental support → bullied online → suicide thoughts = 0.26 × -0.04 × -0.05 = 0.00052

Family economy → parental support → bullied online → depression → suicide thoughts = 0.26 × -0.04 × -0.04 × 0.41 = 0.00017056

Family economy → parental support → pressure → suicide thoughts = 0.26 ×0.03 × 0.05 = 0.00039

Family economy → parental support → pressure → depression → suicide thoughts = 0.26 × 0.03 × 0.44 × 0.41 = 0.00140712

Family economy → depression → suicide thoughts = 0.11× 0.41 = 0.0451

***Parental education to suicide thoughts***

Parental education → pressure → suicide thoughts = 0.03 × 0.05 =0.0015

Parental education → pressure → depression → suicide thoughts = 0.03 × 0.44 × 0.41 = 0.005412

Parental education → parental support → suicide thoughts = -0.08 × 0.13 = -0.0104

Parental education → parental support → bullied others → suicide thoughts = -0.08 × -0.07 × -0.06 = -0.000336

Parental education → parental support → bullied by others → suicide thoughts = -0.08 × -0.03 × -0.04 = 0.000096

Parental education → parental support → bullied by others → depression → suicide thoughts = -0.08 × -0.03 × -0.04 × 0.41 = 0.00003936

Parental education → parental support → bullied online → suicide thoughts = 0.08 × -0.04 × -0.05 =

Parental education → parental support → bullied online → depression → suicide thoughts = 0.08 × -0.04 × 0.41 = -0.001312

Parental education → parental support → pressure → suicide thoughts = 0.08 × 0.07 × 0.05 = 0.00028

Parental education → parental support → pressure → depression → suicide thoughts = 0.08 × 0.07 × 0.44 × 0.41 = 0.00101024

***Friends support to suicide thoughts***

Friends support → pressure → suicide thoughts = 0.21 × 0.05 = 0.0105

Friends support → pressure → depression → suicide thoughts = 0.21 × 0.44 × 0.41 = 0.037884

Friends support → bullied by others → suicide thoughts = -0.37 × -0.04 = -0.0148

Friends support → bullied by others → depression → suicide thoughts = -0.37 × -0.04 × 0.41 =

Friends support → bullied online → suicide thoughts = -0.33 × -0.05 = 0.0015

Friends support → bullied online → depression → suicide thoughts = -0.33 × -0.04 × 0.41 = 0.005412

Friends support → depression → suicide thoughts = 0.15 × 0.41 = 0.0615

***Teacher support to suicide thoughts***

Teacher support → pressure → suicide thoughts = 0.25 × 0.05 = 0.0125

Teacher support → pressure → depression → suicide thoughts = 0.25 × 0.44 × 0.41 = 0.0451

Teacher support → bullied others → suicide thoughts = -0.12 × -0.06 = 0.0072

Teacher support → bullied by others → suicide thoughts = -0.09 × -0.04 = 0.0036

Teacher support → bullied by others → depression → suicide thoughts = -0.09 × -0.04 × 0.41 = 0.001476

Teacher support → bullied online → suicide thoughts = -0.10 × -0.05 = 0.005

Teacher support → bullied online → depression → suicide thoughts = -0.10 × -0.04 × 0.41 = 0.00164

***Parental support to suicide thoughts***

Parental support → pressure → suicide thoughts = 0.07 × 0.44 = 0.0308

Parental support → pressure → depression → suicide thoughts = 0.07 × 0.44 × 0.41 = 0.012628

Parental support → bullied others → suicide thoughts = -0.07 × -0.06 = 0.0042

Parental support → bullied by others → suicide thoughts = -0.03 × -0.04 = 0.0012

Parental support → bullied by others → depression → suicide thoughts = -0.03 × -0.04 ×0.41 = 0.000492

Parental support → bullied online → suicide thoughts = -0.04 × -0.05 = 0.002

Parental support → bullied online → depression → suicide thoughts = -0.04 × -0.04 × 0.41 = 0.000656

Parental support → depression→ suicide thoughts = 0.12 × 0.41 = 0.0492

***Pressure to suicide thoughts***

Pressure → depression → suicide thoughts = 0.44 × 0.41 = 0.1804

***Bullied by others to suicide thoughts***

Bullied by others → depression → suicide thoughts = -0.04 × 0.41 = -0.0164

***Bullied online to suicide thoughts***

Bullied online → depression → suicide thoughts = -0.04 × 0.41 = -0.0164

***Depression***

***Family economy to depression***

Family economy → pressure → depression = 0.08 × 0.44 = 0.0352

Family economy → bullied by others → depression = -0.04 × -0.04 = 0.0016

Family economy → bullied online → depression = -0.05 × -0.04 = 0.002

Family economy → parental support → depression = 0.26 × 0.12 = 0.0312

Family economy → parental support → bullied by others → depression = 0.26 × -0.03 × -0.04 = 0.000312

Family economy → parental support → bullied online → depression = 0.26 × -0.04 × -0.04 = 0.000416

***Parental education to depression***

Parental education → pressure → depression = 0.03 × 0.44 = 0.0132

Parental education → parental support → depression = -0.08 × -0.05 = 0.004

Parental education → parental support → bullied by others → depression = -0.08 × -0.03 × -0.04 = -0.000096

Parental education → parental support → bullied online → depression = -0.08 × -0.04 × -0.04 = -0.000128

***Friends support***

Friends support → pressure → depression = 0.21 × 0.44 = 0.0924

Friends support → bullied by others → depression = -0.37 × -0.04 = 0.0148

Friends support → bullied online → depression = -0.33 × 0.04 = -0.0132

***Teacher support***

Teacher support → pressure → depression = 0.25 × 0.44 = 0.11

Teacher support → bullied by others → depression = -0.09 × -0.04 = 0.0036

Teacher support → bullied online → depression = -0.10 × -0.04 = 0.004

***Parental support to depression***

Parental support → pressure → depression = 0.07 × 0.44 = 0.0308

Parental support → bullied by others → depression = -0.03 × -0.04 = 0.0012

Parental support → bullied online → depression = -0.04 × -0.04 = 0.0016

***Bullied others***

Family economy → parental support → bullied others = 0.26 × -0.07 = -0.0182

Parental education → parental support → bullied others = -0.08 × -0.07 = 0.0056

***Bullied by others***

Family economy → parental support → bullied by others = 0.26 × -0.03 = -0.0078

Parental education → parental support → bullied by others = -0.08 × -0.03 = 0.0024

***Bullied online***

Family economy → parental support → bullied online = 0.26 × -0.04 = -0.104

Parental education → parental support → bullied online = -0.08 × -0.04 = 0.0032

***Pressure***

Family economy → parental support → pressure = 0.26 × 0.07 =0.0182

Parental education → parental support → pressure = -0.08 × 0.07 = -0.0056

**Appendix 7.** Calculation of specific indirect paths between variables for males.

***Self-harm***

***Family economy to self-harm***

Family economy → pressure → depression → self-harm = 0.05 × 0.44× 0.26 = 0.00572

Family economy → bullied others → self-harm = -0.07 × -0.05 = 0.0035

Family economy → bullied others → depression → self-harm = -0.07 × -0.04 × 0.26 = 0.000728

Family economy → bullied by others → self-harm = -0.04 × -0.05 = 0.002

Family economy → bullied by others → depression → self-harm = -0.04 × -0.04 × 0.26 = 0.000416

Family economy → parental support → self-harm = 0.23 × 0.05 = 0.0115

Family economy → parental support → bullied others → depression → self-harm = 0.23 × -006 ×-0.04 × 0.26 = 0.00014352

Family economy → parental support → bullied by others → self-harm = 0.23 × -0.06 × -0.05 = 0.00069

Family economy → parental support → bullied by others → depression → self-harm = 0.23 × -0.06 × -0.04 × 0.26 = 0.00014352

Family economy → parental support → bullied online → self-harm = 0.23 × -0.07 × -0.10 = 0.00161

Family economy → parental support → bullied online → depression → self-harm = 0.23 × -0.07 × -0.07 × 0.29 = 0.00032683

Family economy → depression → self-harm = 0.07 × 0.26 = 0.0182

***Parental education to self-harm***

Parental education → pressure → depression → self-harm = 0.05 × 0.44 × 0.26 = 0.00572

Parental education → parental support → self-harm = -0.05 × 0.05 = -0.0025

Parental education → parental support → bullied others → depression → self-harm = -0.05 × -0.06 × -0.04 × 0.26 = 0.0000315

Parental education → parental support → bullied by others → self-harm = -0.05 × -0.06 × 0.05 = 0.00015

Parental education → parental support → bullied by others → depression → self-harm = -005 × -0.06 × -0.04 × 0.26 = 0.000039

Parental education → parental support → bullied online → self-harm = -0.05 × -0.07 × -0.10 = -0.00035

Parental education → parental support → bullied online → depression → self-harm = -0.05 × -0.07 × -0.07 × 0.26 = 0.0000637

***Friends support to self-harm***

Friends support → pressure → depression → self-harm = 0.23 × 0.44 × 0.26 = 0.026312

Friends support → bullied by others → self-harm = -0.25 × -0.05 = 0.0125

Friends support → bullied by others → depression → self-harm = -0.25 × -0.04 × 0.25 = 0.0025

Friends support → bullied online → self-harm = -0.29 × -0.10 = 0.029

Friends support → bullied online → depression → self-harm = -0.29 × -0.07 × 0.26 = 0.005278

Friends support → depression → self-harm = 0.17 × 0.26 = 0.0442

***Teacher support to self-harm***

Teacher support → pressure → depression → self-harm = 0.25 × 0.44 × 0.26 = 0.0286

Teacher support → bullied others → depression → self-harm = -0.14 × -0.04 × 0.26 = 0.001456

Teacher support → bullied by others → self-harm = -0.09 × -0.05 = 0.0045

Teacher support → bullied by others → depression → self-harm = -0.09 × -0.04 × 0.26 = 0.000936

Teacher support → bullied online → self-harm = -0.10 × -0.10 = 0.01

Teacher support → bullied online → depression → self-harm = -0.10 × -0.07 × 0.26 = 0.00182

***Parental support to self-harm***

Parental support → bullied others → depression → self-harm = -0.06 × -0.04 × 0.26 = 0.000624

Parental support → bullied by others → self-harm = -0.05 × -0.05 = 0.0025

Parental support → bullied by others → depression → self-harm = -0.05 × -0.04 × 0.26 = 0.00052

Parental support → bullied online → self-harm = -0.07 ×-0.10 = 0.007

Parental support → bullied online → depression → self-harm = -0.07 × -0.07 × 0.26 = 0.001274

Parental support → depression→ self-harm = -0.05 × 0.26 = -0.013

***Pressure to self-harm***

Pressure → depression → self-harm = 0.44 × 0.26 = 0.1144

***Bullied others to self-harm***

Bullied others → depression → self-harm = -0.04 × 0.26 = -0.0104

***Bullied by others to self-harm***

Bullied by others → depression → self-harm = -0.04 × 0.26 = -0.0104

***Bullied online to self-harm***

Bullied online → depression → self-harm = -0.07 × 0.26 = -0.0182

***Suicide thoughts***

***Family economy to suicide thoughts***

Family economy → pressure → depression → suicide thoughts = 0.05 × 0.44 × 0.46 = 0.01012

Family economy → bullied others → depression → suicide thoughts = -0.07 × -0.04 × 0.46 = 0.001288

Family economy → bullied by others → suicide thoughts = -0.04 × -0.05 =0.002

Family economy → bullied by others → depression → suicide thoughts = -0.04 × -0.04 × 0.46 = 0.000736

Family economy → parental support → suicide thoughts = 0.23 × 0.08 = 0.0115

Family economy → parental support → bullied others → depression → suicide thoughts = 0.23 × -0.06 × -0.04 × 0.46 = 0.00025392

Family economy → parental support → bullied by others → suicide thoughts = 0.23 × -0.06 × -0.05 = 0.00069

Family economy → parental support → bullied by others → depression → suicide thoughts = 0.23 × -0.05 × -0.04 × 0.46 = 0.0002116

Family economy → parental support → bullied online → suicide thoughts = 0.23 × -0.07 × -0.07 = 0.001127

Family economy → parental support → bullied online → depression → suicide thoughts = 0.23 × 0.07 × -0.07 × 0.46 = -0.00051842

Family economy → depression → suicide thoughts = 0.07 × 0.46 = 0.0322

***Parental education to suicide thoughts***

Parental education → parental support → suicide thoughts = -0.05 × 0.08 = -0.004

Parental education → parental support → bullied others → depression → suicide thoughts = -0.05 × -0.06 × -0.04 × 0.46 = 0.0000552

Parental education → parental support → bullied by others → suicide thoughts = -0.05 × -0.06 × -0.05 = -0.00015

Parental education → parental support → bullied by others → depression → suicide thoughts = -0.05 ×-0.03 × -0.04 = -0.00006

Parental education → parental support → bullied online → suicide thoughts = 0.05 × -0.07 × -0.07 = 0.000245

Parental education → parental support → bullied online → depression → suicide thoughts = 0.05 × -0.07 × -0.07 × 0.46 = 0.0001127

***Friends support to suicide thoughts***

Friends support → pressure → depression → suicide thoughts = 0.23 × 0.44 × 0.46 = 0.046552

Friends support → bullied by others → suicide thoughts = -0.25 × -0.05 = 0.0125

Friends support → bullied by others → depression → suicide thoughts = -0.25 × -0.04 × 0.46 = 0.0046

Friends support → bullied online → suicide thoughts = -0.29 × -0.07 = 0.0203

Friends support → bullied online → depression → suicide thoughts = -0.29 × -0.07 × 0.46 = 0.009338

Friends support → depression → suicide thoughts = 0.17 × 0.46 = 0.0782

***Teacher support to suicide thoughts***

Teacher support → pressure → depression → suicide thoughts = 0.25 × 0.44 × 0.46 = 0.0506

Teacher support → bullied others → depression → suicide thoughts = -0.14 × -0.04 × 0.46 = 0.002576

Teacher support → bullied by others → suicide thoughts = -0.09 × -0.05 = 0.0045

Teacher support → bullied by others → depression → suicide thoughts = -0.09 × -0.04 × 0.46 = 0.001656

Teacher support → bullied online → suicide thoughts = -0.10 × -0.07 = 0.007

Teacher support → bullied online → depression → suicide thoughts = -0.10 × -0.07 × 0.46 = 0.00322

***Parental support to suicide thoughts***

Parental support → bullied others → depression → suicide thoughts = -0.06 × -0.04 × 0.46 = 0.001104

Parental support → bullied by others → suicide thoughts = -0.05 × -0.05 = 0.0025

Parental support → bullied by others → depression → suicide thoughts = -0.05 × -0.04 × 0.46 = 0.00092

Parental support → bullied online → suicide thoughts = -0.07 × -0.07 = 0.0049

Parental support → bullied online → depression → suicide thoughts = -0.07 × -0.07 × 0.46 = 0.002254

Parental support → depression → suicide thoughts = 0.11 × 0.46 = 0.0506

***Pressure to suicide thoughts***

Pressure → depression → suicide thoughts = 0.44 × 0.46 = 0.2024

***Bullied others to suicide thoughts***

Bullied others → depression → suicide thoughts = -0.04 × 0.46 = -0.0184

***Bullied by others to suicide thoughts***

Bullied by others → depression → suicide thoughts = 0.04 × 0.46 = 0.0184

***Bullied online to suicide thoughts***

Bullied online → depression → suicide thoughts = -0.07 × 0.46 = - 0.0322

***Depression***

***Family economy to depression***

Family economy → bullied others → depression = -0.07 × -0.04 = 0.0028

Family economy → bullied by others → depression = -0.04 × -0.04 = 0.0016

Family economy → parental support → depression = 0.23 × 0.11 = 0.0253

Family economy → parental support → bullied others → depression = 0.23 × -0.06 × -0.04 = 0.000552

Family economy → parental support → bullied by others → depression = 0.23 × -0.05 × -0.04 = 0.00046

Family economy → parental support → bullied online → depression = 0.23 × -0.07 × -0.07 = 0.001127

***Parental education to depression***

Parental education → parental support → depression = -0.05 × 0.11 =-0.0055

Parental education → parental support → bullied others → depression = -0.05 × -0.06 × -0.04 = -0.00012

Parental education → parental support → bullied by others → depression = -0.05 × -0.05 × -0.04 = -0.0001

Parental support → parental support → bullied online → depression = -0.05 × -0.07 × -0.07 = -0.000245

***Friends support***

Friends support → pressure → depression = 0.23 × 0.44 = 0.1012

Friends support → bullied by others → depression = -0.25 × 0.04 = -0.01

Friends support → bullied online → depression = -0.29 × -0.07 = 0.0203

***Teacher support***

Teacher support → pressure → depression = 0.25 × 0.44 = 0.11

Teacher support → bullied others → depression = -0.14 × -0.04 = 0.0056

Teacher support → bullied by others → depression = -0.09 × -0.04 = 0.0036

Teacher support → bullied online → depression = -0.10 × -0.07 = 0.007

***Parental support to depression***

Parental support → bullied others → depression = -0.06 × -0.04 = 0.0024

Parental support → bullied by others → depression = -0.05 × -0.04 = 0.002

Parental support → bullied online → depression = -0.07 × -0.07 = 0.0049

***Bullied others***

Family economy → parental support → bullied others = 0.23 ×-0.06 = -0.0138

Parental education → parental support → bullied others = -0.05 × -0.06 = 0.003

***Bullied by others***

Family economy → parental support → bullied by others = 0.23 × -0.05 = -0.0115

Parental education → parental support → bullied by others = -0.05 × -0.03 = 0.0015

***Bullied online***

Family economy → parental support → bullied online = 0.23 × -0.07 = -0.0161

Parental education → parental support → bullied online = -0.05 × -0.07 = 0.0035
